# “No level up!”: no effects of video game specialization and expertise on cognitive performance

**DOI:** 10.3389/fpsyg.2014.01337

**Published:** 2014-11-28

**Authors:** Fernand Gobet, Stephen J. Johnston, Gabriella Ferrufino, Matthew Johnston, Michael B. Jones, Antonia Molyneux, Argyrios Terzis, Luke Weeden

**Affiliations:** ^1^Psychological Sciences, University of LiverpoolLiverpool, UK; ^2^Department of Psychology, Swansea UniversitySwansea, UK; ^3^Department of Psychology, Brunel UniversityUxbridge, UK

**Keywords:** change detection task, expertise, flanker task, transfer, video game playing

## Abstract

Previous research into the effects of action video gaming on cognition has suggested that long term exposure to this type of game might lead to an enhancement of cognitive skills that transfer to non-gaming cognitive tasks. However, these results have been controversial. The aim of the current study was to test the presence of positive cognitive transfer from action video games to two cognitive tasks. More specifically, this study investigated the effects that participants' expertise and genre specialization have on cognitive improvements in one task unrelated to video gaming (a flanker task) and one related task (change detection task with both control and genre-specific images). This study was unique in three ways. Firstly, it analyzed a continuum of expertise levels, which has yet to be investigated in research into the cognitive benefits of video gaming. Secondly, it explored genre-specific skill developments on these tasks by comparing Action and Strategy video game players (VGPs). Thirdly, it used a very tight experiment design, including the experimenter being blind to expertise level and genre specialization of the participant. Ninety-two university students aged between 18 and 30 (*M* = 21.25) were recruited through opportunistic sampling and were grouped by video game specialization and expertise level. While the results of the flanker task were consistent with previous research (i.e., effect of congruence), there was no effect of expertise, and the action gamers failed to outperform the strategy gamers. Additionally, contrary to expectation, there was no interaction between genre specialization and image type in the change detection task, again demonstrating no expertise effect. The lack of effects for game specialization and expertise goes against previous research on the positive effects of action video gaming on other cognitive tasks.

## Introduction

Transfer—the extent to which skills generalize—is an important theoretical concept that has serious practical implications. In a classic article, Thorndike and Woodworth (1901) propounded their theory of “identical elements,” according to which transfer from a first domain to a second domain is possible only when the components of the skills required in each domain overlap. For example, a pianist can use their knowledge of music theory to understand a violin concerto, and a mathematician will understand the differential equations of an economics paper better than a person without background in mathematics. But even in these cases, transfer is far from perfect; for example, the pianist will not be able play the violin concerto itself without extensive additional training.

### Far-transfer

In line with Thorndike and Woodworth's (1901) hypothesis, most theories of expertise predict that transfer from one domain to another (*far-transfer*) will be difficult. This is particularly the case for theories based on the notion that expertise in great part relies on domain-specific perceptual knowledge [e.g., chunking theory (Chase and Simon, 1973) and template theory (Gobet and Simon, 1996)]. While perceptual knowledge enables fluid behavior in the original domain, it is of little use in other domains as it does not match the new environment. Research on chess has provided considerable support for this prediction. Chess players' perceptual skills do not extend to visual memory for shapes (Waters et al., 2002), nor do their planning capabilities transfer to the Tower of London, a task measuring executive function and planning (Unterrainer et al., 2011). Moreover, contrary to widespread belief, there is no robust empirical evidence that playing chess improves scholastic abilities (Gobet and Campitelli, 2006).

One of the rare domains in which evidence of far transfer has been found is playing action video games (e.g., Green et al., 2009)^1^. Repeated playing of this kind of game has been reported to lead to improvements in perceptual and attentional processes and to reduce reaction time in other tasks where one must be both fast and accurate (e.g., Green et al., 2009; Bavelier et al., 2012).

One of the main advantages proposed to be the result of habitual action video game playing is that of a more efficient attentional system. For example, Chisholm et al. (2010) compared action vs. non-action video game players (VGPs) on an attentional capture task where participants searched for a target that could appear in isolation or with a salient task-irrelevant distractor. Action VGPs showed faster reaction times to detect targets and a reduced effect of distractor interference, leading the authors to conclude that the action video gamers had better top-down attentional control, with the consequence that they spend less time processing irrelevant distractors. Consistent with this result, Hubert-Wallander et al. (2011) found that, compared with non-action gamers, action gamers demonstrate superior visual selective attention as measured in a visual search task, with the greatest benefit occurring at the highest cognitive loads (largest search arrays). Additional evidence comes from neuroimaging where differences in brain activation support the idea that long-term video game playing impacts on cortical functioning. For example, using functional magnetic resonance imagery (fMRI), Bavelier et al. (2012) compared a group of action and non-action video gamers on a task involving locating a target stimulus under conditions of increasing distractor load. In addition to overall faster reaction times, compared to the non-video game playing group, the VGPs showed little increase in the level of activation in a network of fronto-parietal sites as distractor load increased. This fronto-parietal network has commonly been associated with attentional processing (Ptak, 2012). These data were taken to suggest that the VGPs were more efficient in their allocation of attentional resources such that the cortical sites deploying attention were able to filter out the distracting information more easily and therefore showed less load dependent increases in cortical activity, supporting the behavioral finding of Chisholm et al. (2010). The proposed superior attentional resource allocation of VGPs (e.g., Hubert-Wallander et al., 2011; Bavelier et al., 2012), that may be at the heart of the observed enhancement of stimulus processing and reduced distractor interference, has now been observed in a number of experiments examining video game expertise based improvements in spatial selective attention (Green and Bavelier, 2003; Feng et al., 2007; Spence et al., 2009), distractor inhibition (Chisholm et al., 2010; Hubert-Wallander et al., 2011; Mishra et al., 2011; Bavelier et al., 2012), enhanced image search (Dye et al., 2009) and target detection (Castel et al., 2005; Dye et al., 2009).

As detailed above, a number of reports using different attentional tasks have suggested that VGPs have an improved ability to “filter out” unnecessary or irrelevant stimuli partly through the enhancement of attentional functioning (Chisholm et al., 2010; Bavelier et al., 2012). One task that has been used successfully to test VGPs proposed advantage at distractor filtering directly is the Flanker Compatibility Task (Eriksen and Eriksen, 1974; Bavelier et al., 2012). The Flanker Task requires participants to ignore salient laterally presented distractors while making responses to a centrally presented target stimulus. Mishra et al. (2011) employed a flanker task to examine whether there was any neuroelectrophysiological evidence of VGP showing enhanced distractor inhibition. The results showed that, behaviourally, VGPs were better able to ignore flanking items competing for attention with a central stimulus than nVGPs and that this increase in behavioral performance was associated with a greater P300 component in the ERP. The P300 electrophysiological component has been associated with perceptual discrimination and decision-making (Picton, 1992; Mishra et al., 2011). These data, together, were taken to support the hypothesis that VGP were better at filtering out the distractor stimuli leading to improved perceptual decision making. Lavie (1995) also reported that through extensive game play VGPs gain the ability to identify task-irrelevant flankers before further processing stimuli. This indicates that VGPs possess an enhanced capability to logically filter information for relevance before attempting to ignore distractors, rather than trying to process everything at once as nVGPs do.

An experimental task that is homologous to the task requirements of many action video games is the change detection task. In the change detection task, participants are asked to monitor a visual display for a small change that they indicate finding via a keypress response. For example, Clark et al. (2011) found that VGPs display a superior ability to spot changes when presented with rapidly alternating sets of images. In this study, 35 participants were presented with an unedited/edited image cycle switching at 4 Hz. The image cycle repeated until the participants indicated via a mouse click that they had spotted the edited element by clicking the image in the position they thought believed contained the image edit. Video game players performed better than nVGPs, replicating previous work on attentional improvements in VGP (e.g., Green et al., 2009), and there were also strategic changes in their search patterns. Compared with nVGP, the VGP showed broader search strategies, further supporting the view that VGPs develop top-down processing.

It follows from the above arguments that, if video-game expertise leads to the observed enhanced attentional and perceptual processing, then it should be possible to train nVGP using video games and observe an improvement in their cognitive functioning. Green and Bavelier (2003) recruited two groups of participants that had no history of video gaming; one group was then trained on a fast-paced action game (*Medal of Honor*) and the other on a slow-paced puzzle game (*Tetris*). After a period of 10 h training (1 h a day over 10 days), compared with the *Tetris* group, participants trained on *Medal of Honor* displayed better Useful Field-of-View (Ball et al., 1988), that is they had an enhancement in their ability to search for and identify cued targets. It was also found that the *Medal of Honor* trainees showed a reduced attentional blink (Raymond et al., 1992), i.e., a reduction in the window of attentional “blindness” that occurs after detecting or recognizing the first of two temporally close visual stimuli (Green and Bavelier, 2003; Feng et al., 2007; Bailey et al., 2010).

While intriguing, the research on the cognitive benefits of video game playing has been criticized on several grounds. Boot et al. (2011) note that experts and novices have different expectations about their performance, which is likely to affect their behavior due to demand characteristics. They also observe that playing video games might affect the kind of strategies that are being used rather than basic perceptual or cognitive capacities. Finally, as some studies have failed to find differences between VGPs and nVGPs, the literature might suffer from a file-drawer problem. Kristjánsson (2013) note that, in many training studies, the control groups do not improve their performance on the tasks of interest, as one would expect, based on the extensive literature on learning, given the test-retest methodology used. In addition, the results might be affected by gender differences, as it is difficult to find expert female VGPs. Both Boot et al. (2011) and Kristjánsson (2013) note the necessity to carry out independent replications.

#### Near transfer

Research has also investigated whether transfer occurs between sub-disciplines of the same field (*near transfer*). For example, do physicians specializing in neurosurgery generalize their skills when solving problems from pediatrics, or do chess players specializing in specific openings (i.e., the first moves of the game) maintain their skill level when making decisions in board positions in which they are not specialized?

Several studies have addressed this issue in medicine (Rikers et al., 2002), political science (Chiesi et al., 1979), and the design of experiments (Schunn and Anderson, 1999). The pattern of results suggests that experts fall back on general heuristics when they cannot use domain-specific knowledge. Emphasizing the role of general problem-solving methods, these studies also highlight the role of domain-specific patterns and methods, as clearly some degree of expertise is lost when domain-specific methods are replaced by domain-general one. While these studies compared individuals of the same level of expertise, Bilalić et al. (2009) compared individuals of different skill levels. They took advantage of several features of chess: chess skill is precisely and quantitatively measured by the Elo rating; chess players enjoy trying to find the best move in a chess position; and chess players specialize in different openings, which makes it relatively easy to find players who have the same strength (as measured by their Elo points) but who have different specialized opening knowledge.

Bilalić et al. compared the performance, in both a memory and problem solving task, of players who specialized in two different chess openings. In addition to positions coming from these two types of defense, they also used neutral positions (positions difficult to classify with respect to the opening they came from). The players were Candidate Masters, Masters, and International Masters/Grandmasters. The results were dramatic. With only one exception, all players obtained the best results with the positions taken from the openings they specialized in. When confronted with positions outside their domain of specialization, players performed one standard deviation on average below the level shown with positions taken from their domain of specialization.

### Aims of the study

Many studies have investigated the differences between VGPs and nVGPs but there is as yet, to our knowledge, no research establishing whether differing levels of video gaming expertise vary with performance on cognitive tasks. Thus, the first aim of this study was to test the hypothesis that, as the level of expertise increases, task accuracy increases, and reaction times become faster.

In addition, a number of studies compare VGPs who identify as “Action” players to nVGPs, but as yet there has been no research into whether the skills demonstrated by action players cross over into other genres, such as strategy games, or indeed if each genre improves different skills. Data from a consumer survey by the Entertainment Software Association found that action and strategy games proved popular with both console and computer VGPs, and so these two genres were chosen as a variable to test the hypothesis (Entertainment Software Association, 2012).

The second aim of the study was thus to test to what extent different video-game genre specialization tap into different cognitive abilities. Action games typically involve fast-paced gameplay, such as *“Call of Duty: Modern Warfare 3”* (Infinity Ward, 2011), which was the best selling action console video game in 2011 (Entertainment Software Association, 2012). It was predicted that the speed of gameplay will heighten action VGPs speeded response times to stimuli other than those normally responded to in a VG, as shown by Green and Bavelier (2003). Strategy players, however, are predicted to possess a stronger reliance on maintaining accuracy as a gained trait from long-term play where accuracy over response time is key to success. This is because, typically, strategy games require the ability to move and place items in carefully decided places and formations. While often these changes are in response to an in-game target event and can result in swift determination and sequencing of new actions to fulfill a shifting long-term goal, there is less emphasis on rapid direct responding to an appearing target. Games such as “*StarCraft 2: Wings of Liberty*” (Blizzard Entertainment, 2010), the best-selling PC strategy game of 2011 (Entertainment Software Association, 2012), demonstrate the need for this ability, particularly in games with a military basis. As a consequence, it was predicted that strategy players would perform with significantly higher mean accuracy and action players would perform with a significantly faster mean reaction time.

The final aim was to replicate the effect of action video-playing on two tasks: a flanker task (Eriksen and Eriksen, 1974) and change detection task (Clark et al., 2011). In particular the flanker task has shown a mixed pattern of results; a basic flanker task has shown both no effect of expertise (Cain et al., 2012), and effects of expertise only once an additional perceptual load has been added (Green and Bavelier, 2003). In the case of Green and Bavelier (2003) it was argued that the addition of a perceptual load prevented flanker interference in the case of nVGP because, unlike the VGP, there were fewer spare resources to process the distracting flankers. However, it did appear in the original Green and Bavelier (2003) that there was a small advantage for VGP compared to nVGP at low loads. We therefore predict that, using a basic “low-load” flanker task, there should be a smaller flanker effect for VGP compared to nVGP and that this will increase as VG expertise decreases.

## Overall method

### Ethical approval

This study was granted ethical approval from the Brunel University School of Social Sciences ethics board in accordance with the British Psychological Society (BPS) guidelines. All participants gave informed consent and were fully debriefed after the study.

### Pilot study

An online pilot, carried out several months before the main study, asked participants (*N* = 115) to identify the last three action and strategy video games they had played. The *Call of Duty* and *Assassin*'*s Creed* video game series were identified as the most popular action video games, and the *Starcraft* and *FIFA* series were found to be the most popular strategy video games.

### Participants

Ninety-two participants (56 male) aged between 18 and 30 (*M* = 21.25, *SD* = 2.07) were recruited by opportunistic sampling through social networking sites and word of mouth. Most of the participants had filled out the online questionnaire (Pilot study). Each participant was offered a food reward for participating in the study, with a further cash reward incentive (£20) if they achieved the best score on one of the two tasks out of all participants.

### Apparatus

The experiment was run using the E-Prime software package (Psychology Software Tools, Inc., 2008) on a Dell desktop computer running Windows 7, with stimuli presented on a 15 inch Lenovo LCD screen running at a resolution of 640 × 480 pixels at a refresh rate of 60 Hz. Keyboard and mouse responses were collected via a standard keyboard and mouse. Participants were sat approximately 60 cm from the computer screen.

### Design

This study was pseudo-experimental in nature, as the independent variables were not directly manipulated. In both experimental measures, the independent variables were skill (experts, intermediates, novices, and controls) and specialization (action vs. strategy). In some analyses, in order to allow direct comparison with the literature, we used skill with only two levels (VGPs vs. nVGPs).

In order to operationalise the study variables, criteria for each between-subject variable needed to be established. A questionnaire was given at the end of the study. In addition to standard questions such as asking age and gender, information was obtained on the participants gaming habits to allow for allocation of each participant to the levels of the two independent variables (i.e., VGP or nVGP). The following three questions were asked.

- “How many hours a week, on average, do you play video games for? 0–1, 2–5, 6–10, 11–15, 16–20, 21+.” This question was used to allocate participants to either the VGP or nVGP group based on their hours of play. Participants who answered “0–1” were allocated to the nVGP group and any answers above were assigned to the VGP group. Sixty-two VGPs and 19 nVGPs were identified.
- “On average, what percentage of the games that you play do you complete? (By completed, we mean attaining the highest in-game ‘level’ or ‘rank’ or completing the game's storyline. You don't need to include optional missions, achievements or DLC (Downloadable Content).” Participants who answered “76–100%” to this were deemed as Experts (*n* = 22), those who answered “51–75%” were deemed Intermediates (*n* = 24) and those who answered “26–50%” were deemed Novices (*n* = 22). Those who answered “0–25%” were allocated to the Control group (*n* = 24).
- “Would you identify yourself predominantly as an action or strategy video gamer?” This question was used to decide each participant's genre specialization. Thirty six participants identified themselves as predominantly action VGPs, alongside 32 strategy VGPs.

Table 1 presents the frequency of participants for each genre × expertise cell of the design.

**Table 1:** **Participation allocation to the experimental groups (genre specialization and expertise level)**.

**Genre specialization and expertise level**	**Frequency (N)**
Action expert	12
Action intermediate	11
Action novice	13
Action control	12
Action total	48
Strategy expert	10
Strategy intermediate	13
Strategy novice	9
Strategy control	12
Strategy total	44
Total	92

### General procedure

Upon arrival, one of the research team allocated the participant a random number that corresponded to the participant's entry in the pilot database that contained information pertaining to their game playing history and experience. They were then handed over to a second experimenter who ran the experiment and who was blind to the participant's details and questionnaire scoring. This method ensured that both the participant and the second researcher were unaware of the participants' genre or expertise allocation. Participants carried out the two experimental measures that formed the study in a random order. For each measure (described below), the first screen to appear was a set of task instructions (see below for details of each experiments instruction). Once the series of tasks was complete, participants completed the “General Information Sheet,” wherein they answered questions such as age, genre specialization, average weekly hours played etc.

### Data analysis

Outliers were identified as either reaction times or correct responses that were notably outside the general distribution. Boxplots for each data set were analyzed and any outliers SPSS identified were removed. All reaction time analyses were performed using correct only trials.

The experiment comprised two measures, an Eriksen Flanker task and a change detection task. Each of these measures is described below.

## Measure 1: eriksen flanker task

This measure was a modified version of Eriksen and Eriksen's (1974) flanker task. Arrows were used instead of letters, similar to other video gaming studies such as Cain et al. (2012).

### Materials

Congruent and incongruent stimuli were created prior to the start of the experiment by combining arrow stimuli such that the central arrow to which the participant responded was surrounded by equally spaced, directionally congruent stimuli (e.g., < < < or > > >) or directionally incongruent stimuli (e.g., < > < or > < >). The flanker stimuli subtended 8° of visual angle.

### Design

The experiment was a mixed factorial design with the within subjects factor being Congruence and between-subjects factors of Expertise Level, Genre Affiliation and “Video Game Players vs. Non-Video Game Players.” The dependent variables in this task were reaction time and percent correct accuracy.

### Procedure

Participants completed 24 congruent and 24 incongruent trials (trial order randomized) in two blocks of equal number (i.e., 24 trials per block). On each trial the participant viewed a centrally presented fixation cross for 500 ms that was replaced by either a congruent or incongruent trial image. Participants viewed each trial image and were asked to indicate in which direction the central arrow using the arrow keys on the keyboard. Each trial remained onscreen until the participant made a key press. The next trial immediately followed.

## Measure 2: change detection task

### Materials

Images were sourced from Google Image Search and were edited using Adobe Photoshop CS5 (Adobe Systems, 2010). Based on the pilot study, which provided information on the most commonly played action and strategy games, images from *Call of Duty* and *StarCraft* were chosen. As both games are part of a much larger series of games, the most recent versions of each franchise were used (*StarCraft II: Wings of Liberty*; Blizzard Entertainment, 2010) and *Call of Duty: Black Ops 2* (Treyarch, 2012). All trial images were scaled such that they subtended 26° of visual angle.

### Design

As it used both expertise and specialization as independent variables, this experiment had the same design as described in Bilalić et al. (2009). Players of different skill levels and specialized with the video games *Call of Duty* or *StarCraft*, as well as a control group of non-players, were presented with images from these two games in addition to non-video game related (defined as “neutral”) stimuli.

The task itself was an adapted form of Clark et al. (2011). There were 13 trials in total, the first of which was a practice trial and was not included in later analyses. Three repeated measures conditions were used: Call of Duty, StarCraft and Landscape (Control) with each condition consisting of all those trials containing the images derived from those games or scenes. There were four images in each condition.

### Procedure

**Figure 1:**
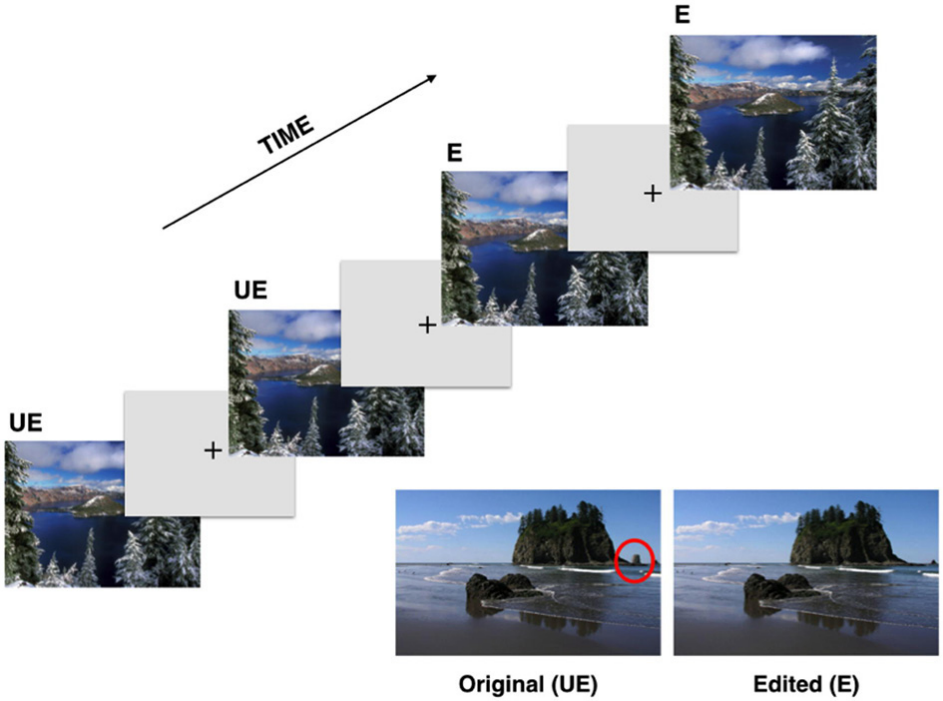
**One cycle of a sample Change Detection trial showing a Landscape (Control) image**. The sequence involved two presentations of the unedited image prior to two presentations of an edited image. An example of an unedited and associated edited image is shown in the bottom right corner: The feature missing in the edited version of the image is indicated by the red circle on the original image.

Participants fixated a centrally presented cross for 4000 ms prior to the start of the change detection task image presentation. The first, unedited (UE), image was then presented for 240 ms, followed by a blank gray screen for 80 ms. A second image, identical to the first, would then appear for a period of 240 ms before being replaced by a blank gray screen for 80 ms. The process would then repeat but with the edited (E) version (identical save for a change in a single image feature) of the same image, i.e., the sequence appeared as UE, UE, E, E, UE, UE, E, E… This cycle repeated until the participant responded by pressing the spacebar on the computer keyboard (see Figure 1).

On detection of a change, the participant ceased the trial via keypress (spacebar) and then reported both the location and nature of the perceived change to the experimenter who remained with the participant in the room during data collection. The participant then pressed the spacebar again in order to trigger the next trial presentation.

## Results

### Measure 1: eriksen flanker task

Prior to analysis, one outlier was removed for failing to comply with task instruction. Data were analyzed using a mixed ANOVA to determine the effect of specialization and skill (between-subjects) with performance on congruent and incongruent trials (within-subjects).

#### Accuracy

Analyses indicated a main effect of congruence, *F*_(1, 83)_ = 27.90, *p* < 0.001, η^2^_*p*_ = 0.25, *f* = 0.58. The Congruent (Same) condition (*M* = 23.69, *SD* = 0.69) had a higher average score than the Incongruent (Distractor) condition (*M* = 18.90, *SD* = 8.38). This effect was not qualified by participant expertise, *F*_(3, 83)_ = 0.57, *p* = 0.64, η^2^_*p*_ = 0.02, *f* = 0.14, or genre, *F*_(1, 83)_ = 2.55, *p* = 0.11, η^2^_*p*_ = 0.03, *f* = 0.18. No interaction was found between congruence, expertise and genre, *F*_(3, 83)_ = 2.00, *p* = 0.12, η^2^_*p*_ = 0.07, *f* = 0.27.

#### Reaction time

Analyses showed a main effect of congruence on reaction time, *F*_(1, 73)_ = 53.31, *p* < 0.001, η^2^_*p*_ = 0.42, *f* = 0.85; overall, a lower mean reaction time was demonstrated in the Congruent (Same) condition (*M* = 433.85, *SD* = 70.47) than the Incongruent (Distractor) condition (*M* = 529.46, *SD* = 155.82). This effect was not qualified by participant expertise, *F*_(3, 73)_ = 0.42, *p* = 0.74, η^2^_*p*_ = 0.02, *f* = 0.14, or genre, *F*_(1, 73)_ = 1.71, *p* = 0.59, η^2^_*p*_ = 0.006, *f* = 0.08. No interaction was found between congruence, expertise and genre, *F*_(3, 73)_ = 1.71, *p* = 0.17, η^2^_*p*_ = 0.07, *f* = 0.27.

#### VGPs vs. nVGPs

**Figure 2:**
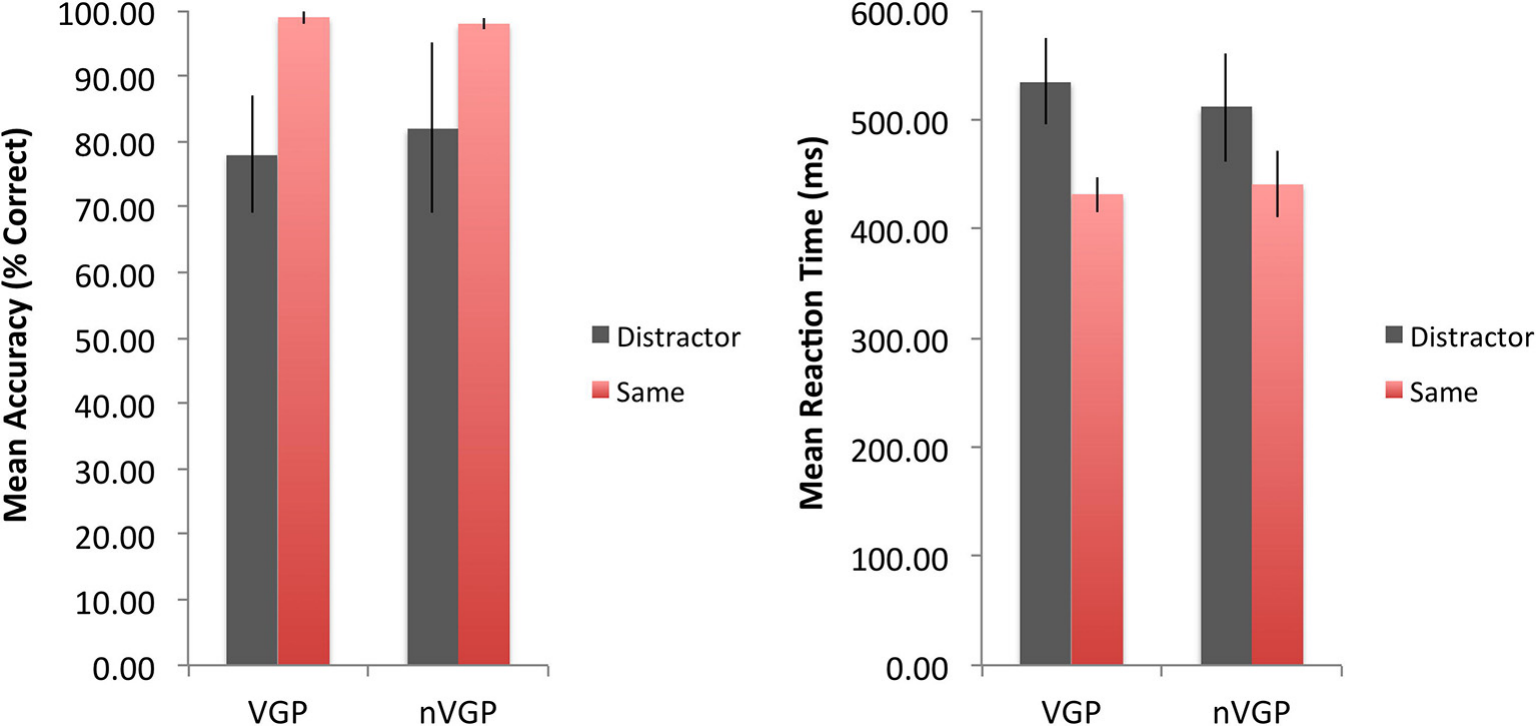
**Mean accuracy (left), reaction times (right) and the associated 95% confidence intervals for the VGP and nVGP from the Eriksen Flanker Task**.

In order to attempt to replicate previous research using this task, we also carried out analyses where the participants were allocated to only two groups (VGPs and nVGPs). nVGPs were identified as any participant who played, on average, less than 1 h of either console or computer video games per week. Figure 2 shows a summary of the reaction time and accuracy data for the flanker task for VGPs and nVGPs.

With respect to accuracy, there was a main effect of congruence on accuracy, *F*_(1, 89)_ = 19.96, *p* < 0.001, η^2^_*p*_ = 0.18, *f* = 0.47, that was not qualified by the players' status [*F*_(1, 89)_ = 0.24, *p* = 0.62, η^2^_*p*_ = 0.008, *f* = 0.09].

With respect to reaction time, both groups performed overall faster in the Congruent (Same) condition (*M* = 433.85, *SD* = 70.47) than the Incongruent (Distractor) condition (*M* = 529.46, *SD* = 155.82). Analysis indicated a main effect of congruence on reaction time, *F*_(1, 79)_ = 32.23, *p* < 0.001, η^2^_*p*_ = 0.29, *f* = 0.64, that was not qualified by the players' status, *F*_(1, 79)_ = 1.12, *p* = 0.29, η^2^_*p*_ = 0.01, *f* = 0.1.

## Measure 2: change detection task

After outliers were removed due to task non-compliance, 85 participants remained from the original 92. A Mixed ANOVA was carried out and outliers were controlled in an identical way to the Flanker Task.

Analysis indicated a main effect of image type on reaction time, *F*_(2, 154)_ = 36.57, *p* < 0.001, *n*^2^_*p*_ = 0.32, *f* = 0.69. Response times were quicker in the Landscape condition (*M* = 9399 ms, *SD* = 5119 ms) than in the Call of Duty condition (*M* = 16,138 ms, *SD* = 7556 ms) and Starcraft condition (*M* = 20,247 ms, *SD* = 10,761 ms). This effect was not qualified by participant expertise [*F*_(6, 154)_ = 1.06, *p* = 0.39, *n*^2^_*p*_ = 0.04, *f* = 0.2] or genre [*F*_(2, 154)_ = 0.57, *p* = 0.57, *n*^2^_*p*_ = 0.01, *f* = 0.1]. No interaction was found between image type, expertise and genre [*F*_(6, 154)_ = 0.27, *p* = 0.95, *n*^2^_*p*_ = 0.01, *f* = 0.1].

**Figure 3:**
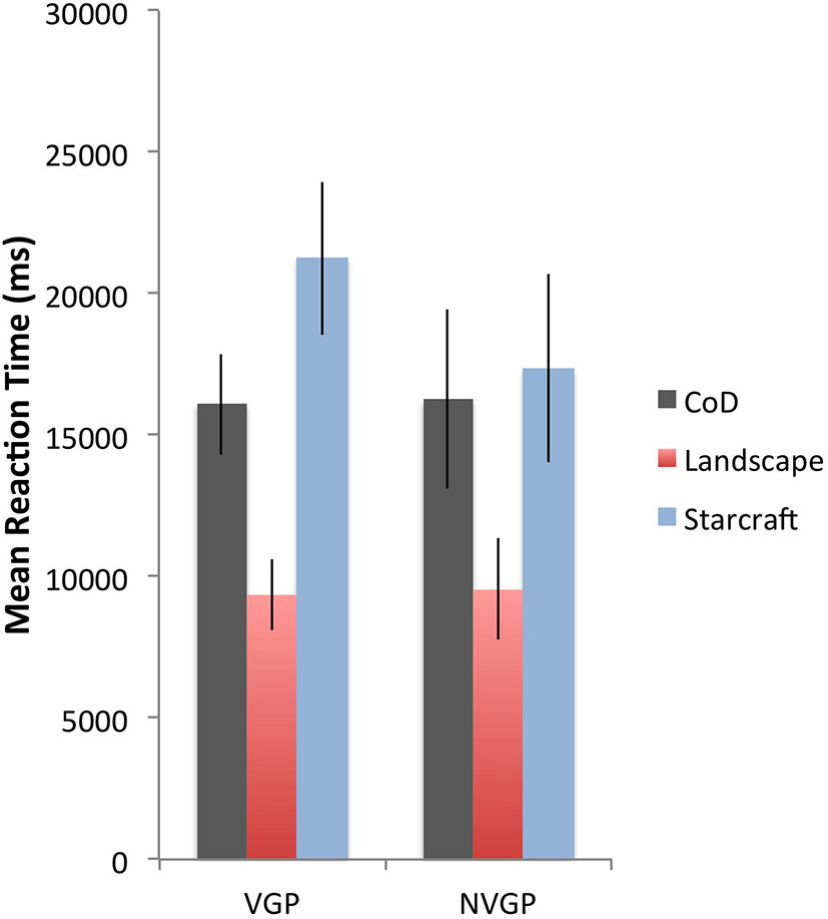
**Mean reaction times along with the associated 95% confidence intervals for VGP and nVGP from the Change Detection Task**.

We also analyzed the data by grouping the participants into players and non-players. Mauchly's Test indicated a violated assumption of sphericity, χ^2^_(2)_ = 21.57, *p* < 0.001, therefore degrees of freedom (*df*) were corrected using Greenhouse-Geisser estimates of sphericity (ε = 0.81). Analysis indicated a main effect of image type on reaction time, *F*_(2, 166)_ = 25.06, *p* < 0.001, *n*^2^_*p*_ = 0.23, *f* = 0.55, that was not qualified by the “Video Game Players vs. Non-Video Game Players” variable [*F*_(2, 166)_ = 1.37, *p* = 0.26, *n*^2^_*p*_ = 0.02, *f* = 0.14]. Figure 3 shows a summary of the reaction time data for the change detection task for VGPs and nVGPs.

## Power summary

One advantage of this study is the relatively large number of participants who were involved. However, to ensure that the absence of expertise effects was not due to a lack of statistical power, we investigated the size of effects we could be expected to find. All calculations are based on a power criterion of 0.8 and a 0.05 alpha level. For Measure 1: Eriksen Flanker task, one could expect to find significant differences of effect sizes for the interaction of congruency and specialization of 0.31 and of 0.36 for the two-way interaction of congruency with skill and for the three-way interaction of congruency with skill and specialization. For Measure 2: Change Detection Task, effect size of 0.25 could be expected to be identified for interactions of image type with specialization and effect sizes of 0.30 for both the two-way interactions of specialization and skill with image type and the three-way interaction of image type, specialization, and skill.

In all cases, the observed effect sizes for game specialization and skill in each of the measures was considerably smaller than the minimum expected detectable effect size even given our relatively large sample size. The power analyses also highlight that given our sample size we could expect to detect small effect sizes.

## Discussion

One of the major conclusions of research into learning and expertise is that transfer from one domain to another is rare and difficult, and happens only when the two domains share components that ask for the same cognitive skills. In recent years, a series of experiments on action video game playing have found that playing this kind of game leads to substantial transfer, in particular with tasks engaging attentional processing. The aim of this study was to replicate this phenomenon with two tasks that had previously been used in the action video-game literature. In addition, the study aimed to use a finer measure of expertise than had been done in the past, and to look at the extent to which skills acquired in a specific VG genre (action or strategy) can be used in a task using material linked to either of the two genres.

In neither task were we able to find any effect of skill or a superiority of the VGPs when compared to the nVGPs. Thus, our study did not support the hypothesis of far transfer, in line with most theories of expertise but in contradiction with previous VG studies. Our failure to replicate previous results cannot be ascribed to a lack of power, as the number of participants (*n* = 92) was high for this kind of study and our design, incorporating different levels of skill, was in principle able to identify subtle skills effects that cannot be found when only two groups are compared (VGPs vs. nVGPs). In addition, the results we obtained in each task were consistent with the results normally obtained in these tasks. For example, we found significantly faster reaction times and a greater number of percent correct accurate trials for the congruent trials compared to the non-congruent trials in the flanker task.

For the flanker task, despite strong congruency effects, the absence of a significantly different interference effect for VGP vs. nVGP is not entirely unexpected, despite our predictions to the contrary. We argued that based on previous work that we might expect a small difference (Green and Bavelier, 2003), especially given our sample size and a finer division of game expertise than previously used. However, this was not the case and, although the null hypothesis cannot be accepted, the absence of a significant interaction of game expertise and congruency does add to a number of results showing that, at low loads there is little difference between the performance on VGP and nVGP on a flanker task (Green and Bavelier, 2003; Cain et al., 2012).

We followed Boot et al.'s (2011) advice of pre-screening participants long before the experiments *per se*, and of asking them to fill in the questionnaire on video game activities at the end of the study. Thus, our procedure minimized demand characteristics. Together with the fact that other studies have failed to find a VGP effect (e.g., Castel et al., 2005; Boot et al., 2008; Murphy and Spencer, 2009), our results are consistent with the possibility that such demands might have played a role in previous research showing VGPs' superiority. However, given that this conclusion is based on null results, further research is needed to validate or invalidate this hypothesis.

Most unexpected was perhaps the failure to find an expertise effect in the change detection task. Near transfer did not occur in spite of the fact that the material used came from the players' domain of expertise (either action or strategy players) and that we based the stimulus choice on the most popular games within each genre, an expected level of increased familiarity. Why is it that the patterns that the players had presumably acquired by playing their favorite game did not enable them to find changes in the stimuli more rapidly? One possible explanation is that the change detection paradigm is unsuitable for detecting domain-specific patterns used for unconscious pattern recognition. An examination of the mean reaction times shows that the average time spent on task was very long (about 20 s on average in the Starcraft condition) and thus is likely to engage more conscious mechanisms. This explanation gains further plausibility given that Gobet et al. (in preparation) did find an interaction between specialism and expertise in a recognition task using a similar design as that used here: action players specializing in the *Call of Duty* and racing players specializing in *Gran Turismo* performed better when dealing with images from their own game.

Our study was not without weaknesses. The measure of video-game expertise and the allocation to a specific genre were based on self-reports, and perhaps it would have been desirable (albeit unpractical) to ask players to play segments of their favorite game to estimate their level. With regard to obtaining a pure measure of specific video game genre benefits a study, such as this, that concentrates on training effects in nVGP (e.g., Green and Bavelier, 2003) may be in a better suited to detect subtle changes in performance. A comparison of preferred gametype may be expected to find subtle differences between players of different games but only when those players partake of a single type of game. Anecdotally, many game players are “poly-gamers” and will play other game types in addition to their preferred category. A training study where nVGP gain experience playing only a single genre of game would therefore be better placed to detect subtle differences between expertise benefits of individual game types. A similar argument can be made to better examine any potential gender differences. The sample here did not allow for a meaningful investigation of potential gender differences and, for the same reasons previously argued to account for poly-gamers, a training study would be ideal. Finally, there were few trials in the change detection task.

In the last year several professional associations and journals have emphasized the need for more replications. However, in spite of previous calls (e.g., Gobet et al., 2004), and partly due to the difficulty of finding experts, research into expertise is rarely replicated. The current paper contributes to this effort of obtaining more robust empirical data.

### Conflict of interest statement

The authors declare that the research was conducted in the absence of any commercial or financial relationships that could be construed as a potential conflict of interest.

## Footnotes

^1^In this study, the term “video game” refers to any published computer or console video game from 1952 to the present day. The term “video game player” (shortened to VGP) refers to any individual who plays these games, and the term “non-video game player” (shortened to nVGP) refers to any individual who partakes in video game play for less than 1 h per week.

## References

[B1] Bailey K. West R. Anderson C. A. (2010). A negative association between video game experience and proactive cognitive control. Psychophysiology 47, 34–42 10.1111/j.1469-8986.2009.00925.x 19818048

[B2] Ball K. Beard B. Roenker D. Miller R. Ball D. (1988). Visual search age and practice. Invest. Ophthalmol. Vis. Sci. 29, 448.

[B3] Bavelier D. Achtman R. L. Mani M. Föcker J. (2012). Neural bases of selective attention in action video game players. Vision Res. 61, 132–143. 10.1016/j.visres.2011.08.007 21864560 PMC3260403

[B4] Bilalić M. McLeod P. Gobet F. (2009). Specialization effect and its influence on memory and problem solving in expert chess players. Cogn. Sci. 33, 1117–1143. 10.1111/j.1551-6709.2009.01030.x 21585497

[B5] Boot W. R. Blakely D. P. Simons D. J. (2011). Do action video games improve perception and cognition? Front. Psychol. 2:226. 10.3389/fpsyg.2011.00226 21949513 PMC3171788

[B6] Boot W. R. Kramer A. F. Simons D. J. Fabiani M. Gratton G. (2008). The effects of video game playing on attention, memory, and executive control. Acta Psychol. 129, 387–398. 10.1016/j.actpsy.2008.09.005 18929349

[B7] Cain M. S. Landau A. N. Shimamura A. P. (2012). Action video game experience reduces the cost of switching tasks. Atten. Percept. Psychophys. 74, 641–647. 10.3758/s13414-012-0284-1 22415446

[B8] Castel A. D. Pratt J. Drummond E. (2005). The effects of action video game experience on the time course of inhibition of return and the efficiency of visual search. Acta Psychol. 119, 217–230. 10.1016/j.actpsy.2005.02.004 15877981

[B9] Chase W. G. Simon H. A. (1973). Perception in chess. Cogn. Psychol. 4, 55–81 10.1016/0010-0285(73)90004-2

[B10] Chiesi H. L. Spilich G. J. Voss J. F. (1979). Acquisition of domain-related information in relation to high and low domain knowledge. J. Verbal Learn. Verbal Behav. 18, 257–273 10.1016/S0022-5371(79)90146-4

[B11] Chisholm J. D. Kingstone A. Hickey C. Theeuwes J. (2010). Reduced attentional capture in action video game players. Attent. Percept. Psychophys. 72, 667–671. 10.3758/APP.72.3.667 20348573

[B12] Clark K. Fleck M. S. Mitroff S. R. (2011). Enhanced change detection performance reveals improved strategy use in avid action video game players. Acta Psychol. 136, 67–72. 10.1016/j.actpsy.2010.10.003 21062660

[B13] Dye M. W. G. Green C. S. Bavelier D. (2009). The development of attention skills in action video game players. Neuropsychologia 47, 1780–1789. 10.1016/j.neuropsychologia.2009.02.002 19428410 PMC2680769

[B14] Entertainment Software Association. (2012). Essential Facts About the Computer and Video Game Industry. Available online at: http://www.theesa.com/facts/pdfs/ESA_EF_2012.pdf (Accessed March 11, 2013)

[B15] Eriksen B. A. Eriksen C. W. (1974). Effects of noise letters upon identification of a target letter in a nonsearch task. Percept. Psychophys. 16, 143–149 10.3758/BF03203267

[B16] Feng J. Spence I. Pratt J. (2007). Playing an action video game reduces gender differences in spatial cognition. Psychol. Sci. 10, 850. 10.1111/j.1467-9280.2007.01990.x 17894600

[B17] Gobet F. Campitelli G. (2006). Education and chess: a critical review, in Chess and Education: Selected Essays from the Koltanowski Conference, ed Redman T. (Dallas, TX: Chess Program at the University of Texas at Dallas), 124–143.

[B18] Gobet F. de Voogt A. J. Retschitzki J. (2004). Moves in Mind. Hove: Psychology Press.

[B19] Gobet F. Patel K. Johnston S., (in preparation). The Effects of Video Game Specialisation on a Recognition Task.

[B20] Gobet F. Simon H. A. (1996). Templates in chess memory: a mechanism for recalling several boards. Cogn. Psychol. 31, 1–40. 10.1006/cogp.1996.0011 8812020

[B21] Green C. S. Bavelier D. (2003). Action video game modifies visual selective attention. Nature 423, 534. 10.1038/nature01647 12774121

[B22] Green C. S. Li R. J. Bavelier D. (2009). Perceptual learning during action video game playing. Topics Cogn. Sci. 2, 202–216. 10.1111/j.1756-8765.2009.01054.x 25163784

[B23] Hubert-Wallander B. Sugarman M. Bavelier D. Green C. S. (2011). Changes in search rate but not in the dynamics of exogenous attention in action videogame players. Attent. Percept. Psychophys. 73, 2399–2412. 10.3758/s13414-011-0194-7 21901575

[B24] Infinity Ward. (2011). Call of Duty: Modern Warfare 3 [Video Game]. Santa Monica, CA: Activision.

[B25] Kristjánsson Á. (2013). The case for causal influences of action videogame play upon vision and attention. Attent. Percept. Psychophys. 75, 667–672. 10.3758/s13414-013-0427-z 23386038

[B26] Lavie N. (1995). Perceptual load as a necessary condition for selective attention. J. Exp. Psychol. Hum. Percept. Perform. 21, 451–468. 10.1037/0096-1523.21.3.451 7790827

[B27] Mishra J. Zinni M. Bavelier D. Hillyard S. A. (2011). Neural basis of superior performance of action videogame players in an attention-demanding task. J. Neurosci. 31, 992–998. 10.1523/JNEUROSCI.4834-10.2011 21248123 PMC6632922

[B28] Murphy K. Spencer A. (2009). Playing video games does not make for better visual attention skills. J. Artic. Support Null Hypothesis 6, 1–20.

[B29] Picton T. W. (1992). The P300 wave of the human event-related potential. J. Clin. Neurophysiol. 9, 456–479. 10.1097/00004691-199210000-00002 1464675

[B30] Ptak R. (2012). The frontoparietal attention network of the human brain: action, saliency, and a priority map of the environment. Neuroscientist 18, 502–515. 10.1177/1073858411409051 21636849

[B31] Raymond J. E. Shapiro K. L. Arnell K. M. (1992). Temporary suppression of visual processing in an RSVP task—an attentional blink. J. Exp. Psychol. Hum. Percept. Perform. 18, 849–860. 10.1037/0096-1523.18.3.849 1500880

[B32] Rikers R. M. J. P. Schmidt H. G. Boshuizen H. P. A. Linssen G. C. M. Wesseling G. Paas F. G. W. C. (2002). The robustness of medical expertise: clinical case processing by medical experts and subexperts. Am. J. Psychol. 115, 609–629. 10.2307/1423529 12516530

[B33] Schunn C. D. Anderson J. R. (1999). The generality/specificity of expertise in scientific reasoning. Cogn. Sci. 23, 337–370 10.1207/s15516709cog2303_3

[B34] Spence I. Yu J. J. Feng J. Marshman J. (2009). Women match men when learning a spatial skill. J. Exp. Psychol. Learn. Mem. Cogn. 35, 1097–1103. 10.1037/a0015641 19586273

[B35] Thorndike E. L. Woodworth R. S. (1901). The influence of improvement in one mental function upon the efficiency of other functions. Psychol. Rev. 9, 374–382. 9866023

[B36] Unterrainer J. M. Kaller C. P. Leonhart R. Rahm B. (2011). Revising superior planning performance in chess players: the impact of time restriction and motivation aspects. Am. J. Psychol. 124, 213–225. 10.5406/amerjpsyc.124.2.0213 21834406

[B37] Waters A. J. Gobet F. Leyden G. (2002). Visuo-spatial abilities in chess players. Br. J. Psychol. 30, 303–311 10.1348/000712602761381402 12519534

